# Autobiographical Implicit Association Test and eye movements: fixations topography enables detection of autobiographical memories

**DOI:** 10.3389/fpsyg.2024.1268256

**Published:** 2024-01-29

**Authors:** Andrea Zangrossi, Liisa Camilla Gatto, Virginia Lanfranchi, Cristina Scarpazza, Miriam Celli, Giuseppe Sartori

**Affiliations:** ^1^Department of General Psychology, University of Padova, Padova, Italy; ^2^Padova Neuroscience Center (PNC), University of Padova, Padova, Italy; ^3^IRCCS S.Camillo Hospital, Venezia, Italy

**Keywords:** autobiographical memory, aIAT, eye movements, fixations, eye-tracking

## Abstract

**Introduction:**

Autobiographical memory is the capacity to recollect memories of personally experienced events. The detection of such memories plays a key role in criminal trials. Among behavioral memory-detection methods, the autobiographical Implicit Association Test (aIAT) has gained popularity for its flexibility and suitability for forensic applications. The aIAT is a reaction time-based methodology aiming to assess whether information about an event is encoded in the respondent’s mind. Here, we introduced the *eye-D* index, a measure based on the topography of fixations while performing the aIAT, as an additional measure to detect autobiographical memories covertly.

**Methods:**

In this study, participants were involved in a mock-crime experiment in which they could act as Guilty or Innocent. One week later all participants underwent the aIAT combined with eye-tracking to investigate the presence of the crime-related memory.

**Results:**

Guilty participants showed a higher number of fixations towards the category labels in the block in which true sentences shared the same response key with crime-related sentences, as compared to the block in which true sentences were paired with sentences describing an alternative version. Innocent participants showed the opposite pattern. This unbalanced allocation of attention to the category labels was quantified by the eye-D index and was found to be highly correlated to the standard aIAT-D index.

**Discussion:**

This suggests that more fixations to the category labels could indicate increased cognitive load and monitoring of response conflicts. These preliminary results highlight eye-tracking as a tool to detect autobiographical memories covertly while performing the aIAT.

## 1 Introduction

Autobiographical memory refers to an individual’s capacity to recollect events that they have personally experienced. Detecting specific individual memories plays an essential role during crime investigations and criminal trials, i.e., to identify whether a suspect possesses the guilty memory. Several memory-detection methods have been designed to identify crime-related memories, such as the Concealed Information Test (CIT) or Guilty Knowledge Test (GKT; Lykken, 1959; Verschuere and Ben-Shakhar, 2011). The shared logic behind these tools is that a “guilty” individual will recognize relevant details of the crime that are unknown to an innocent person (for a review on behavioral lie-detection techniques see Sartori et al., 2018). The recognized details can be identified based on the triggered behavioral response (e.g., reaction times; Sartori et al., 2008; Agosta and Sartori, 2013; Zangrossi et al., 2015), psychophysiological (e.g., skin conductance response; Verschuere and Ben-Shakhar, 2011; Ben-Shakhar, 2012), and neural responses (e.g., P300; Rosenfeld et al., 2008; Gamer and Berti, 2012).

Among behavioral techniques, a promising tool is the autobiographical Implicit Association Test (aIAT; Sartori et al., 2008), a modified version of the Implicit Association Test (Greenwald et al., 1998) that aims to assess which of two autobiographical events is true (Sartori et al., 2008). In summary, the aIAT consists of stimuli from four categories: two logical categories with sentences that are objectively true or false for the respondent at the time of testing (e.g., “I’m in front of the computer” vs. “I’m climbing a mountain”), and two alternative versions of the construct under investigation, only one being true (e.g., “I spent my holidays in Paris” vs. “I spent my holidays in London”). The respondent is asked to categorize the sentences by pushing keys located on the left or the right of a keyboard (usually “A” and “L”). Each key is associated with category labels that are always shown in boxes on the left or the right side of the screen, respectively. For instance, for the abovementioned example, the screen could show the label “PARIS” on the left (indicating that sentences related to spending holidays in Paris should be associated with the left key) and “LONDON” on the right (indicating that sentences related to spending holidays in London should be associated to the right key). The task is structured into five categorization blocks: three simple categorization blocks where only sentences belonging to one category are shown (i.e., only TRUE/FALSE or PARIS/LONDON sentences), and two critical blocks characterized by a double categorization (i.e., sentences belonging to both categories are presented). In the combined categorization blocks, sentences belonging to different categories share the same response key. For instance, one could be required to push the left key both for true and Paris-related sentences, and the right key both for False and London-related sentences. Pairing true sentences with sentences about real autobiographical memories should lead to faster RTs (this condition is called “Compatible block”), conversely, when true sentences share the same key with an alternative version of the event (i.e., not matching memory content) RTs are longer (“Incompatible block”).

The logic underlying IAT-based methods is based on the so-called compatibility effect. According to the creators of the original IAT (Greenwald et al., 1998), this effect arises from the conceptual link between the target and attribute categories (Kinoshita and Peek-O’Leary, 2005). In the case of the air, it pertains to the conceptual association between one of the two versions of the investigated construct (e.g., committing a crime) and a logical category (e.g., True). From a memory detection perspective, this suggests that one of the construct’s versions aligns with autobiographical memory, potentially indicating that information about the crime is encoded in the respondent’s episodic memory. The compatibility effect strength can be individually computed using the IAT-D Index (Greenwald et al., 2003). This index is used to measure the strength of the association between true statements and the two versions of the episode, thus allowing us to infer which version matches individual episodic memory and whether the result obtained can be considered reliable.

The IAT has been tested in various contexts, including past and future intentions, white lies and underlying intentions, mock crimes, holidays, drug consumption, driver’s licenses, flashbulb memories, and whiplash malingering (Agosta and Sartori, 2013; Curci et al., 2015; Zangrossi et al., 2015) as well as in real cases of suspected crime-related amnesia (Zago et al., 2023). While several independent studies have confirmed the reliability of the aIAT method (e.g., Hu et al., 2012; Hu and Rosenfeld, 2012; Freng and Kehn, 2013; Takarangi et al., 2013; Shidlovski et al., 2014; Takarangi et al., 2015; Verschuere and Kleinberg, 2017), some investigations have highlighted wide variability in classification accuracy (Verschuere et al., 2009; Vargo and Petróczi, 2013; Morgan et al., 2014), with low performance when the aIAT was not used to test a specific autobiographical event, such as the case of drug abuse (Vargo and Petróczi, 2013).

One of the limitations of the aIAT, especially for real-world forensic applications, is that it requires a certain amount of compliance from the respondent, who sometimes can deliberately try to fake their behavioral performance to avoid being discovered. While some specific faking strategies (e.g., deliberate slowing of responses) can be reliably detected (Cvencek et al., 2010; Agosta et al., 2011b), other strategies may remain undiscovered. Moreover, other factors can potentially affect test performance, such as a false alibi (Dhammapeera et al., 2020), source confusion, and familiarity (Takarangi et al., 2013, 2015).

Due to the abovementioned limitations which apply both to the aIAT and other behavioral memory-detection techniques, the combination with additional covert indices is desirable to promote accuracy and reliability, especially for forensic applications. Eye movements have the potential to be an ideal candidate for this purpose since they occur at rapid timescales and could reflect moment-to-moment changes in memory processing (Kragel and Voss, 2022), thus supporting the detection of the true autobiographical memory trace.

Several studies (Andrews and Coppola, 1999; Rayner et al., 2007; Castelhano and Henderson, 2008; Boot et al., 2009; Poynter et al., 2013) suggest that some eye movement parameters might represent an endogenous signature of the observer, relatively independent from visual input. Accordingly, in a previous study we showed that spatiotemporal eye-movement dynamics during free-viewing of scenes were highly similar to those while viewing a blank screen (without any meaningful visual information) (Zangrossi et al., 2021), and were related to stable properties of brain activity at rest (Celli et al., 2022).

Aside from stable intrinsic characteristics, literature shows that eye movements can also reveal ongoing cognitive processing and are sensitive to cognitive load in reading tasks (Rayner, 2012; Mahanama et al., 2022). A recent study (Ogawa et al., 2021) tested the use of pupillometry during the administration of the aIAT and found that in the incompatible block, the pupil diameter was larger as compared to the compatible block, thus showing that pupillometry can serve as a viable measure of the compatibility effect elicited by the aIAT. It is well-known that pupil dilation is correlated with cognitive load (Krejtz et al., 2018), which is the main driver of the slowdown of RTs introduced by the aIAT. Aside from pupil dilation, other eye-movements-based parameters can also give information about the compatibility effect in aIAT, such as the spatial distribution of fixations. Indeed, from a cognitive perspective, fixations refer to information processing (Just and Carpenter, 1980) making them a potential candidate for measuring cognitive load. Some studies have related cognitive load to fixation duration (Nuthmann et al., 2010; Liu et al., 2022) with higher cognitive load eliciting longer fixations.

Conversely, we hypothesized that increased cognitive load in the aIAT would lead to a higher number of fixations to critical information presented on the screen (i.e., category labels). This is in line with some studies investigating the prediction of cognitive performance in RTs tasks using machine-learning models based on eye-tracking features (Arsalidou et al., 2021; Bachurina et al., 2022). These studies showed that the number of fixations was the best predictor of cognitive performance. An alternative hypothesis could suggest a role of fixations duration, which can also be considered as a potential predictor of cognitive performance in RTs-based tasks (Arsalidou et al., 2021; Bachurina et al., 2022), despite being outperformed by the number of fixations. We believe that fixations duration is not a suitable parameter for the detection of cognitive load in the aIAT, since this task is explicitly grounded on temporal pressure (i.e., categorize sentences as fast as you can), thus putting strong constraints on fixation duration. For this reason, we hypothesized that increased RTs (i.e., increased cognitive load) will not lead to increased fixation duration in the Incompatible block, but rather to a higher number of fixations to critical information presented on the screen (i.e., category labels), thus highlighting a key role of fixation topography.

Furthermore, in forensic scenarios, an uncooperative examinee may attempt to employ countermeasures during testing. In this context, fixation duration could be more susceptible to countermeasures, such as a deliberate effort to extend fixation time in the Compatible block. This is in line with previous works on aIAT’s faking attempts (Cvencek et al., 2010; Agosta et al., 2011b). Conversely, achieving accurate responses in the Incompatible block relies on scrutinizing category labels (i.e., checking category associations).

In the present paper, we aimed to investigate whether the aIAT compatibility effect can be detected covertly by eye movements. To this end, we focused on the topographical distribution of fixations during the aIAT and we hypothesized that in the Incompatible block, participants would produce more fixations to the boxes showing category labels (i.e., AOIs) as compared to the Compatible block. This would allow to compute an eye-movements-based aIAT-D counterpart which could help to covertly capture the compatibility effect.

## 2 Materials and methods

### 2.1 Participants

We recruited a sample of *N* = 68 participants (17 males; mean age = 23.89 and SD = 2.29 years) with normal or corrected-to-normal vision, all of them being native Italian speakers, as part of a larger project on the influence of crime-related variables on autobiographical memory detection. The sample size was based on previous studies on similar topics using the same paradigm (Dhammapeera et al., 2020) and was designed to have a power > 0.8 to detect effect sizes *d* > 0.7 The sample comprised students at the University of Padova recruited through social media platforms or by advertisements. All participants provided written informed consent for their participation, and the study was approved by the Ethical Committee for Psychological Research of the University of Padova. Participants were randomly assigned to one of two conditions – Innocent (*N* = 35) and Guilty (*N* = 33) – which required different actions to be performed in a mock crime (see below).

### 2.2 Apparatus

In this study, we utilized a Windows 10 PC along with a screen-based eye tracker with a sampling rate of 600 Hz (TOBII Pro Spectrum, Stockholm, Sweden) for the binocular recording of eye movements. The aIAT stimuli were presented on the native 23.8-inch Tobii Pro Spectrum screen (EIZO [Ishikawa, Japan] FlexScan EV2451) with a pixel resolution of 1920 × 1,080 (52.8 × 29.7 cm). The screen was located approximately 65 cm in front of the participant (visual angle 9.22°), who was sitting on a steady and comfortable chair in a dimly lit room. Before the aIAT administration, a 9-point calibration procedure was performed to ensure data accuracy. Recalibrations were made if deemed necessary by the examiner. The aIAT was implemented using PsychoPy (Peirce et al., 2019).

### 2.3 Procedure

The experiment consists of two sessions, with the second one taking place 1 week apart, lasting 15–20 min and 30 min, respectively. The first session included a mock-crime procedure in which the participants had to act according to their experimental condition (either Innocent or Guilty). Specifically, participants in the Guilty group were asked to enter a room indicated on a map (a laboratory in the basement of the Department of Psychology at the University of Padova) and destroy a photograph depicting a crime (i.e., sexual assault). To this end, we extracted frames from the play *“Extremities”* written by William Mastrosimone staged by Teatro Due – Parma in 2009 and directed by Bruno Armando; in the photo are the actors Alessandro Averone and Paola De Crescenzo (reproduced with the author’s permission). This choice was made because this study is part of a broader project aimed at investigating how different degrees of a crime (e.g., sexual assault vs. sexual harassment) impact memory detection. However, this is not discussed in the present study. On the other hand, participants in the Innocent group [similarly to Hu et al. (2015)] were instructed to go to the same area just to write their participation code (i.e., a number) on a sheet of paper hanging on the wall and were not aware of any mock-crime taking place.

One week later, all participants were asked to come back to the laboratory for the administration of an aIAT about the memory of the action performed during the first session. Moreover, simultaneous eye-movement recording was performed.

#### 2.3.1 aIAT structure

**Figure 1:**
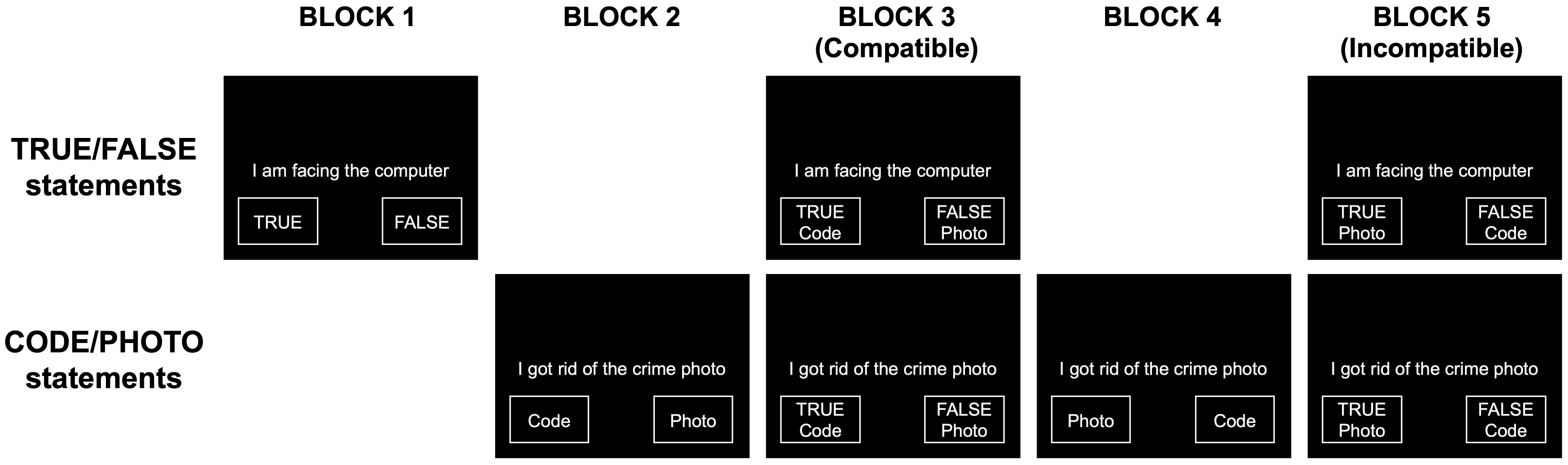
aIAT task structure. The figure shows the typical aIAT structure with different sentence categories being involved in different blocks.

The aIAT was structured in 5 blocks (see Figure 1) and included sentences belonging to the logical dimension True/False or sentences describing two alternative versions of the memory under evaluation (writing participant’s code vs. destroying the photo of the crime; see Table 1 for the sentences used). Specifically, in block 1 (20 trials), participants classified stimuli as True (left key) or False (right key). In block 2 (20 trials), participants classified sentences along the critical dimension: Code vs. Photo. They were asked to press the left key to classify sentences related to writing the participant’s code on a sheet of paper (Code) and to press the right key to classify sentences about destroying the photo of the crime (Photo). In block 4 (20 trials), participants were requested to perform a reversed classification as compared to block 2: left key for Photo and right key for Code.

**Table 1:** aIAT sentence stimuli.

Category	Sentence	Ground truth
True	I am in Padua I am in a room with a computer I am conducting a psychology experiment I am sitting on a chair I am facing the computer	True for all participants
False	I am climbing a mountain I am in Rome I am having lunch at a restaurant I am playing soccer I am in a shop	False for all participants
Photo	I tore the photo of the crime I got rid of the crime photo I eliminated the only evidence of the crime I destroyed the evidence of the crime I disposed of the evidence of the crime	True for participants in the Guilty group; False for participants in the Innocent group
Code	I wrote my code on a sheet of paper I wrote a code on a sheet of paper I jotted down my code on a sheet of paper I wrote on a poster hanging on the wall I inputted the code on a sheet of paper	True for participants in the Innocent group; False for participants in the Guilty group

Note that sentences were presented in Italian in the experiment since all participants were native Italian speakers.

The double categorization blocks (3 and 5) were composed of 60 trials each. We defined “Compatible block” as a block in which the association between categories matched the innocent version (i.e., “True” associated with “Code” and “False” associated with “Photo”). Conversely, the “Incompatible block” was the one associating sentences that should be linked only in the mind of a guilty participant (i.e., “True” associated with “Photo,” “False” associated with “Code”).

Importantly, labels indicating category names were displayed on the computer screen for the entire duration of the experiment (see Figure 1).

Notably, the position of the Compatible and Incompatible blocks (blocks 3 and 5) was randomized across participants.

### 2.4 Analysis

#### 2.4.1 Data preprocessing

Before any further analysis, RTs shorter than 150 ms or longer than 10,000 ms were discarded, consistently with previous papers (Zangrossi et al., 2015) Moreover, RTs related to incorrect responses were replaced with the mean RTs of correct trials with the addition of 600 ms (Greenwald et al., 2003). For eye-movement data, raw data were processed to detect fixations using a velocity-based threshold algorithm (Engbert and Kliegl, 2003) with a detection threshold lambda = 15, which has been considered robust across several testing conditions (Stuart et al., 2019). Moreover, we identified the boxes where the category labels were presented during the whole aIAT (see Figure 1 for a representation of the graphical structure of the aIAT) as Areas of Interest (AOIs), and we classified each fixation as falling inside or outside the AOIs. Notably, for the present study, we did not distinguish between left and right AOIs, but we counted the total number of fixations within both AOIs.

#### 2.4.2 Mixed-effects models on RTs

Four nested Generalized Linear Mixed-Effects regression models were built utilizing RTs as the dependent variable, subject as the random effect factor, and Block (Compatible vs. Incompatible) and Group (Innocent vs. Guilty) as fixed effects regressors. Specifically, the null model, referred to as Model 0, solely contained the random effect. Model 1 introduced the Block as a predictor, Model 2 further incorporated the contribution of the Group, and Model 3 also included the interaction between the Block and the Group. Notably, to approximate reaction times (RTs), a Gamma-family function was selected as the link function, since this is a suitable model for RTs approximation (Whelan, 2008; Baayen and Milin, 2010). Models were compared through a Likelihood Ratio test (LRT), as well as using unbiased indices of goodness of fit, i.e., the Akaike Information Criterion (AIC; Akaike, 1987; Hurvich and Tsai, 1989) and the Bayesian Information Criterion (BIC; Schwarz, 1978). These indices are recommended to select the model with the best balance between likelihood and parsimony (i.e., the number of predictors), hence accounting for the risk of overfitting. The best model is the one that minimizes AIC and BIC.

#### 2.4.3 Quantification of the compatibility effect using RTs and eye-movements

To quantify the compatibility effect in RTs, we computed the standard aIAT-D index (Greenwald et al., 2003). This index is a type of D score in which the average RTs of the Compatible block are subtracted from the average RTs of the Incompatible block, then this difference is divided by the pooled standard deviation (SD) of subject RTs in both blocks. Since the RTs associated with incorrect responses are replaced with the mean RTs of that block plus a 600-ms penalty, this index is also called D600. In the present study, a larger positive score is taken as an indication of the association between the action of writing the participant code on a sheet, and the truth.

As for eye movements, we computed an index that could be considered the eye-movements counterpart of the aIAT-D, which we called *eye-D.* This index is based on the difference in the individual proportion of fixations (%) located within the AOIs (i.e., the boxes where the category labels are shown during the aIAT) between critical blocks. We also considered other candidate eye-movement metrics, namely fixation duration and time to first fixation within the AOIs (TFF), however, the best candidate resulted to be the proportion of fixations to the AOIs. The eye-D was computed through the following formula:


$$\mathrm{eye}-D=\frac{\begin{array}{l} \mathrm{fixations}\;\mathrm{AOIs}\;\left(\mathrm{Incompatible}\right)-\\ \mathrm{fixations}\;\mathrm{AOIs}\;\left(\mathrm{Compatible}\right)\; \end{array}}{\mathrm{pooled}\;SD\;\mathrm{of}\;\mathrm{fixations}\;\mathrm{AOIs}\;\mathrm{across}\;\mathrm{groups}\;\mathrm{and}\;\mathrm{blocks}}$$

In summary, this index represents the individual tendency to pay attention to the category labels differently according to the level of difficulty elicited by the critical blocks.

All analyzes were performed using R software (R Core Team, 2019). For generalized linear mixed-effect models, we used the R package lme4 (Bates et al., 2015).

## 3 Results

### 3.1 The compatibility effect in reaction times

**Figure 2:**
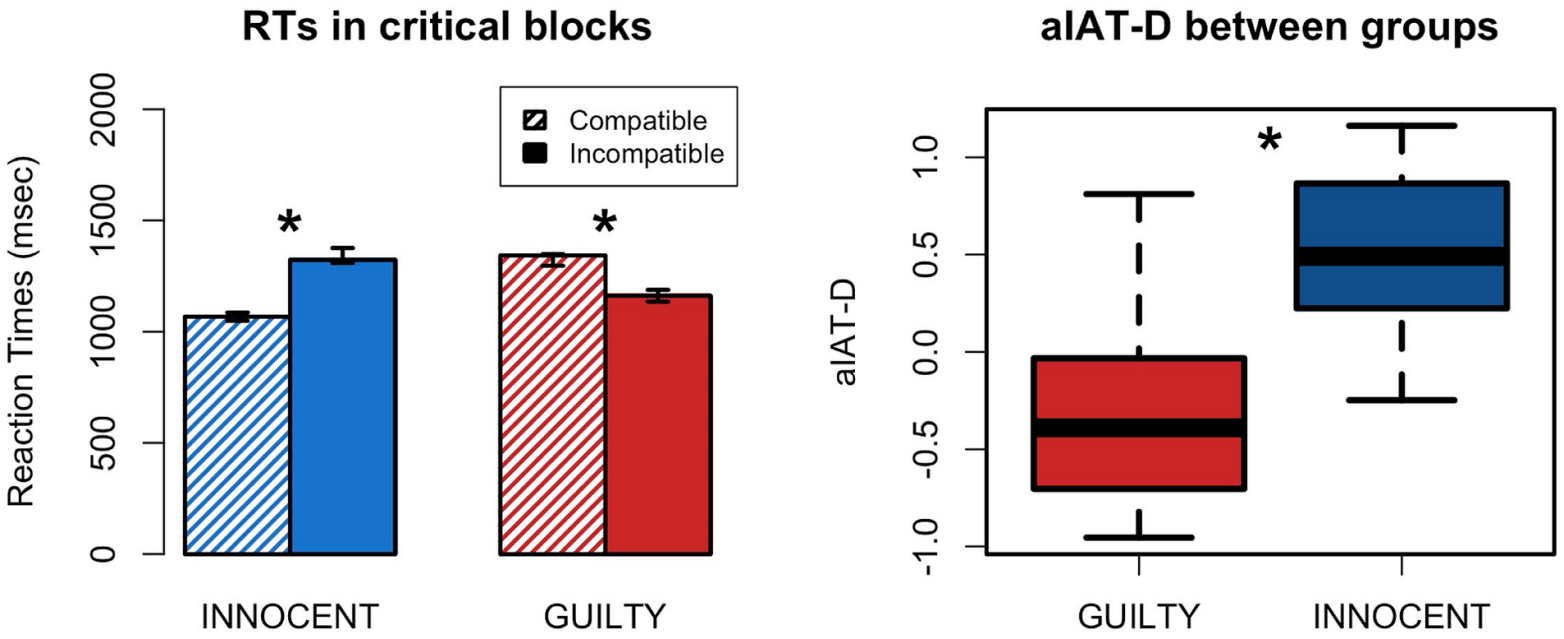
aIAT compatibility effect in Guilty and Innocent group. Left: barplot showing the mean RTs obtained by Guilty and Innocent in the critical blocks. Error bars represent 95% C.I. Right: comparison between Groups in the aIAT-D index; *, significant difference.

To test which predictor best explained RTs during the aIAT we built four nested models, which were then compared using a LRT. The statistical assumptions for generalized linear regression were met. The LRT showed that Model 3 was the best in describing RTs in our experiment (Table 2). This result indicates that the addition of the interaction between Block and Group significantly improved the model explaining RTs during the aIAT. The best model showed a main effect of Block (χ^2^ [1] = 20.72, *p* < 0.001) but not of Group, as well as a significant interaction Block*Group (*χ*^2^ [1] = 548.84, *p* < 0.001). This result suggests that participants in the Guilty group did not show a generalized slowing down of RTs which is sometimes referred to as a *guilty mindset.* On the other hand, Guilty and Innocent participants show a different pattern of RTs in the two critical blocks (Figure 2). Specifically, post-hoc least-squares means showed that Innocents made higher RTs in the Incompatible as compared to the Compatible block (z = 20.08, p < 0.001), while Guilty showed the opposite pattern (*z* = −12.9, *p* < 0.001). This result was further confirmed by computing an individual index to quantify the compatibility effect, i.e., the aIAT-D, which showed significant differences between Guilty and Innocent participants (*t* [63.37] = −8.497, *p* < 0.001; Figure 2 right).

**Table 2:** Model comparison through Likelihood Ratio test (LRT).

Model	AIC	BIC	*χ*^2^ (Df)	*p*-value
Model 0: random effect	16,223	16,244	–	
Model 1: random effect + Block	16,205	16,233	20.65 (1)	<0.001
Model 2: random effect + Block + Group	16,206	16,242	0.167 (2)	0.683
Model 3: random effect + Block + Group + Block:Group	15,670	15,712	538.82 (2)	<0.001

In all models, RTs were the dependent variable. AIC, Akaike Information Criterion; BIC, Bayesian Information Criterion.

### 3.2 The compatibility effect in eye movements

**Figure 3:**
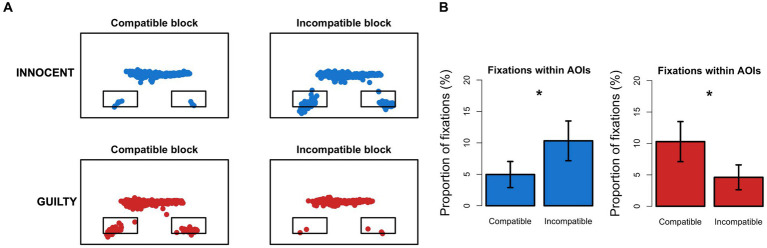
Fixations into the AOIs. **(A)** Distribution of fixations (colored dots) on the screen during the aIAT in two exemplars Innocent (blue) and Guilty (red) participants. Small rectangles represent the AOIs where the category labels were shown during the blocks. **(B)** Barplot representing the proportion of fixations falling into the AOIs in the Compatible and Incompatible blocks. Color mapping is the same as in **(A)** (Innocent = blue; Guilty = red). Error bars represent 95% C.I. of the mean; *, significant difference.

We first counted the number of fixations within each AOI (i.e., boxes with the category labels) in the Compatible and Incompatible blocks. Then, tested whether the proportion of fixations (%) within the AOIs was different between critical blocks and whether this difference was modulated by group (Figure 3). To this end, we ran repeated measures mixed ANOVA with the proportion of fixations within AOIs as the dependent variable, the group as between factor, and the block as within factor. We found a significant interaction between group and block (*F* [1,66] = 30.371, *p* < 0.001) which suggested that the difference between Compatible and Incompatible blocks in the proportion of fixations within AOIs was modulated by the group. Specifically, Innocent participants made significantly more fixations to the AOIs in the Incompatible block as compared to the Compatible block (*t* [34] = −3.66, *p* < 0.001), while Guilty participants showed the opposite pattern (*t* [32] = 4.19, *p* < 0.001). We also ran the same procedure with fixation duration and TFF as dependent variables. The ANOVA on fixation duration showed no significant interaction between group and block, neither for fixations within AOIs (*F* [1,66] = 2.09, *p* = 0.15) nor for fixations outside AOIs (*F* [1,66] = 3.04, *p* = 0.086). The main effect of block (*F* [1,66] = 0.85, *p* = 0.36 and *F* [1,66] = 1.27, *p* = 0.27, respectively) and group (*F* [1,66] = 0.35, *p* = 0.56 and *F* [1,66] = 0.76, *p* = 0.39) were also not significant. When employing TFF we found a trend to significance for the main effect of the block (*F* [1,52] = 3.93, *p* = 0.053), while group (*F* [1,52] = 0.29, *p* = 0.59), and block*group interaction (*F* [1,52] = 1.34, *p* = 0.25) were not significant. Notably, the different degrees of freedom in this analysis are caused by 15 subjects who did not show saccades directed to the AOIs in at least one of the two critical blocks.

**Figure 4:**
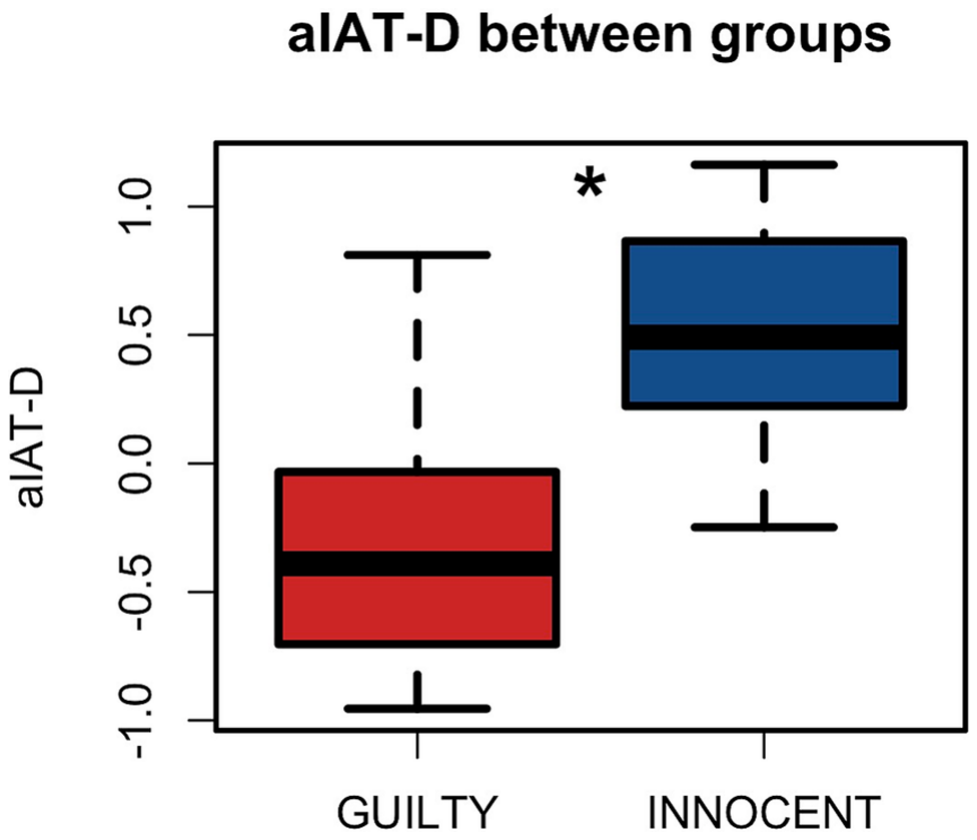
Group comparison in the eye-D index. The difference between groups on individual eye-D values. eye-D represents the eye-movements-based quantification of the Compatibility effect measured by the standard aIAT-D index. *significant difference.

Furthermore, we computed individual values of eye-D as described before and we found that significant difference between Innocent and Guilty participants (*t* (65.85) = −5.53, *p* < 0.001), with Innocent participants showing average positive eye-D values, while Guilty participants showed negative eye-D values (Figure 4). This is in line with the aIAT-D index and suggests that for Innocent participants the Incompatible block (i.e., TRUE/Photo) was more difficult than the Compatible block (i.e., TRUE/Code), and thus it required more fixations to the AOIs, while the Guilty participants showed the opposite pattern.

Taken together, these results show that the compatibility effect in the aIAT task was mainly captured by the higher number of saccades directed to the AOIs (i.e., more fixations within the AOIs), without impacting TFF and fixation duration.

### 3.3 The relation between aIAT-D and eye-D

**Figure 5:**
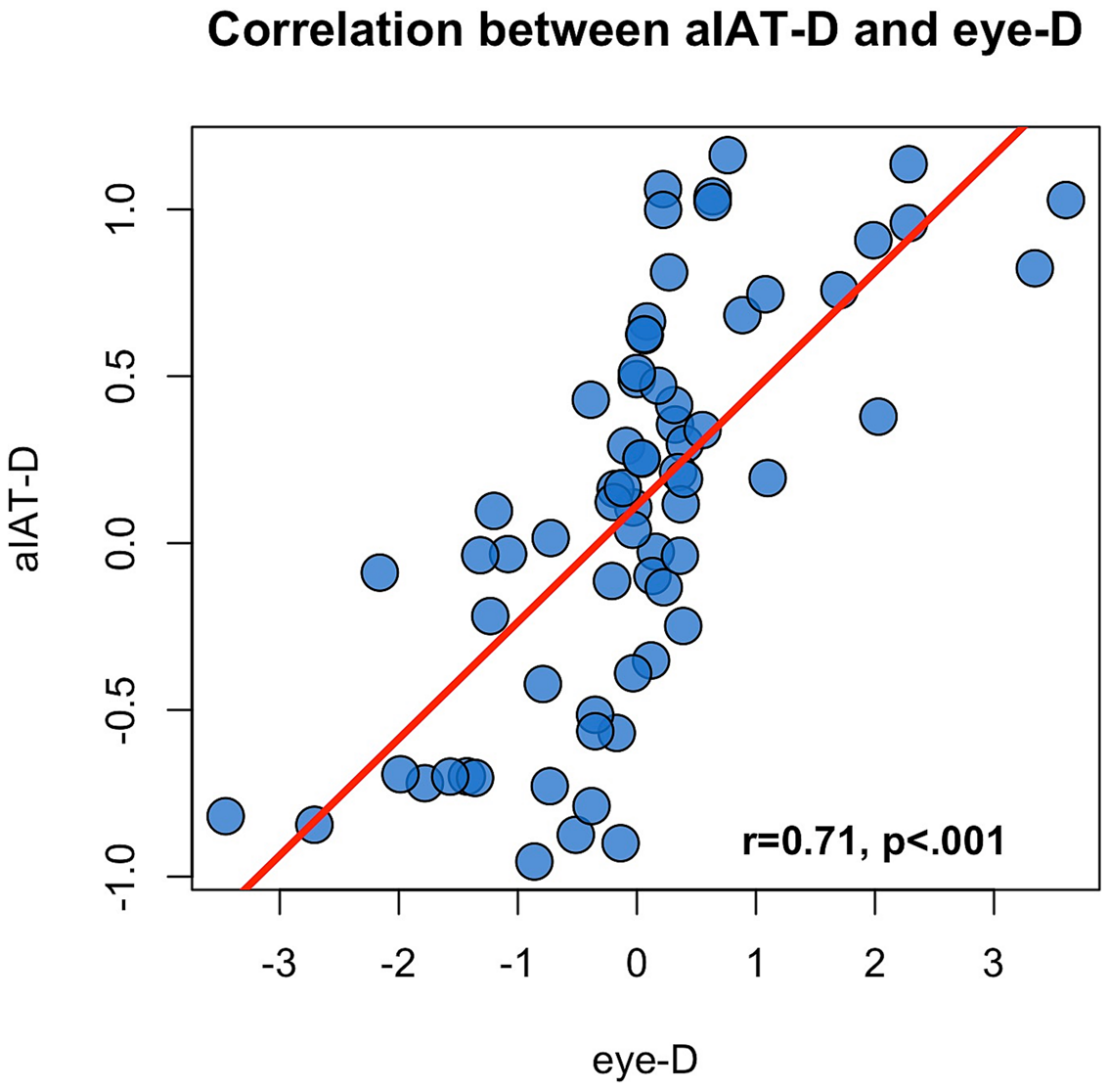
Correlation between aIAT-D and eye-D.

Finally, we wanted to investigate the relation between aIAT-D and eye-D and to compare these indices in the identification of Guilty participants. We found that aIAT-D and eye-D are highly correlated (*r* = 0.71, *p* < 0.001; Figure 5) which is in line with the idea that both indices are designed to capture the Compatibility effect from the RTs and eye-movements perspective, respectively.

Furthermore, we compared the accuracy in the identification of Guilty and Innocent participants between aIAT-D and eye-D. Specifically, we used a cut-off of 0 to identify Guilty participants as those with negative aIAT-D values and Innocents as those with positive aIAT-D values. We found that the aIAT-D correctly identified 26 out of 33 Guilty and 33 out of 35 Innocents (accuracy = 86.7%; specificity = 94.3%; sensitivity = 78.8%). Then we applied the same logic to the eye-D index, and we found that this measure correctly identified 24 out of 33 Guilty and 27 out of 35 Innocents (accuracy = 75%; specificity = 77%; sensitivity = 72.3%). In 47 out of 68 cases (69%), the aIAT-D and the eye-D showed a matching classification. Interestingly, the combination of both indices allowed for improved classification sensitivity. Indeed, classifying as Guilty all participants having either a positive aIAT-D or eye-D allowed us to correctly identify 28 out of 33 Guilty, thus improving sensitivity (85%).

## 4 Discussion

People can manipulate overt responses when asked to refer about a crime-related memory. A century-old question is whether the real memory trace is still detectable less consciously and more automatically. Eye movements have the potential to be an ideal candidate for this purpose since they occur on the same timescale as many neural processes and thus could reflect moment-to-moment changes in memory processing (Kragel and Voss, 2022). To the best of our knowledge, only one study (Ogawa et al., 2021) investigated the potential of eye-tracking as an additional measure of the compatibility effect elicited by the aIAT. Specifically, in this study, the authors recorded a specific measure, i.e., pupil dilation, during aIAT administration and found that in the incompatible block, the pupil diameter was larger as compared to the compatible block. This result is in line with studies describing pupil dilation as an index of cognitive load, with pupil diameter increasing with problem difficulty (for a review see van der Wel and van Steenbergen, 2018).

In the present study, we focused on spatial properties of eye movements in a mock-crime experiment, to investigate whether the aIAT compatibility effect might be covertly measured from the pattern of fixations while performing the task. In other words, we investigated whether the different task difficulty elicited by the Compatible and Incompatible blocks can be detected independently from overt responses (i.e., RTs). Our results confirmed the presence of the compatibility effect. Specifically, participants in the Innocent group showed significantly lower RTs in the condition where true statements and statements referred to writing their code on a paper shared the same motor response (Compatible block), as compared to the condition where the same response button was shared between true statements and crime-related statements (Incompatible block). Conversely, participants in the Guilty group showed the opposite pattern of RTs in the two blocks, with longer RTs in the Compatible block. These results were confirmed both by Generalized Linear Regression models at the group-level and by individual aIAT-D values which showed significant differences between groups. Turning to eye movements, we hypothesized that the increased cognitive load related to the abovementioned response conflict could lead to more numerous fixations to the boxes where the category labels (which encode critical information to provide correct responses) are presented, which we identified as our Areas of Interest (AOIs). Thus, we introduced an index called eye-D which was designed to represent the eye-movements counterpart of the aIAT-D, since it was designed to capture the compatibility effect independently from RTs. The eye-D index is computed from the proportion of fixations directed to the AOIs in the two critical blocks of the aIAT, which could be considered as an attempt to suppress automatic responses by repeatedly checking the correct response key (i.e., the information provided within the AOIs which are either on the left or on the right). We found significant differences in eye-D values between Guilty and Innocent participants which showed an opposite pattern of fixation distribution into the AOIs. Specifically, participants in the Innocent group made more fixations towards the AOIs in the Incompatible block (True/Photo – False/Code) since the association between categories did not match their actual memory trace (*“I wrote my code on a sheet of paper”*). On the other hand, participants in the Guilty group showed a higher number of fixations to the AOIs in the Compatible block (True/Code – False/Photo) which was not in line with their memory (*“I destroyed the photo of the crime”*). Our findings suggest that the topography of fixations, while a subject is performing the aIAT, can covertly detect the compatibility effect as measured by the standard aIAT-D index.

One possible explanation for this result relates to the concept of cognitive load. Indeed, in standard aIAT, the compatibility effect is quantified using the pattern of RTs that are higher in the Incompatible block, thus suggesting increased cognitive load. This increased response latency was not mirrored by increased fixation durations, nor by time to first fixation within the AOIs (TFF), but only by an increased number of fixations to critical information showed on the screen (i.e., boxes with category labels). Interestingly, the sensitivity in the identification of guilty participants was higher (85%) when considering both the aIAT-D and the eye-D, as compared to the aIAT-D alone (78%). This suggests that the heightened cognitive demand in the Incompatible block results in more fixations on the category labels (AOIs). This behavior may be seen as an effort to enhance response accuracy by repeatedly verifying the association between the category label and the corresponding key to press. Accordingly, the increased number of fixations towards the AOIs could relate to response monitoring. In a mock-crime study, Hu et al. (2015) found that guilty participants performing the CIT showed larger Late Posterior Negative slow-wave (LPN) for probe stimuli. The enlarged LPN is thought to reflect response monitoring (Johansson and Mecklinger, 2003) and might be caused by the conflict arising from the inhibition of the automatic recognition process and the selection of the correct response key. Accordingly, a previous study (Agosta et al., 2011a) found a larger Late Positive Component (LPC) in the Compatible as compared to the Incompatible block. Enhancement of LPC is thought to relate to recognition memory (Friedman and Johnson, 2000; Munte et al., 2000) and is associated with a pattern of shorter RTs (Johnson et al., 1998, 2003), in line with the aIAT effect. Taken together, these results suggest that memory detection techniques can be enriched by the application of covert indices that could improve the applicability of these tools in forensic practice. Among these, eye movements have several advantages since they are relatively easy to obtain, even using PC cameras, and can be applied covertly, as compared to electrophysiological techniques. Further studies on this topic could include a larger set of eye-movement features to deal with the issue of covert memory detection in a multivariate fashion. Moreover, the analysis of eye movements could benefit from the use of less structured tasks, e.g., the Concealed Information Test (CIT). One open question in the memory detection literature is whether easy-to-obtain behavioral markers of a memory trace can be made more resistant to faking attempts. The ability of the aIAT to resist faking attempts has been studied and led to controversial results (Cvencek et al., 2010; Agosta et al., 2011b). For instance, guilty subjects who have been imagining an alibi for some time (voluntarily or not) show an average D-IAT index close to zero (Dhammapeera et al., 2020). Moreover, Hu et al. (2015) found that guilty participants who were explicitly asked to suppress their memory of the crime to fake the aIAT and CIT showed a reduction of aIAT-D score as well as of P300 amplitude, which is linked to the conscious recollection of episodic memories (Paller et al., 1995; Vilberg et al., 2006; Rugg and Curran, 2007). This suggests that a voluntary attempt to suppress crime-related memories can critically impact the overt aIAT behavioral response. However, as mentioned above, guilty participants in this experiment could still be identified by their enlarged LPN, probably reflecting the conflict between top-down voluntary memory suppression and automatic recognition processes (Hu et al., 2015).

We propose that eye movements offer a promising avenue for covertly identifying concealed memories when used alongside traditional behavioral methods. Additional research is required to explore how this integration can bolster the resilience of behavioral tools against feigning efforts. Deliberate attempts to deceive the aIAT (as well as other behavioral memory-detection techniques) should theoretically heighten cognitive load and induce response conflicts. In this perspective, eye-tracking can be integrated with behavioral methods as a covert technique to render such deception more discernible. Indeed, this combination can potentially reveal concealed aspects of attentional processes that may remain unidentified through behavioral performance alone, enhancing the overall identifiability of target memories.

## Data availability statement

The data supporting the conclusions of this article will be made available by the corresponding author upon reasonable request, without undue reservation.

## Ethics statement

The studies involving humans were approved by Ethical Committee for Psychological Research of the University of Padova. The studies were conducted in accordance with the local legislation and institutional requirements. The participants provided their written informed consent to participate in this study.

## Author contributions

AZ: Conceptualization, Data curation, Formal analysis, Methodology, Supervision, Writing – original draft, Writing – review & editing. LG: Data curation, Investigation, Writing – original draft. VL: Data curation, Investigation, Writing – original draft. CS: Supervision, Writing – review & editing. MC: Writing – review & editing. GS: Funding acquisition, Supervision, Writing – review & editing.
